# EVALUATING INSTRUMENTS FOR ASSESSING HEALTHSPAN: A MULTI-CENTER CROSS-SECTIONAL STUDY IN THE COMPANION DOG

**DOI:** 10.1093/geroni/igac059.2936

**Published:** 2022-12-20

**Authors:** Frances Chen, Tarini Ullal, Jessica Graves, Ellen Ratcliff, Alexander Naka, Brennen McKenzie, Tennery Carttar, Michael LaCroix-Fralish

**Affiliations:** Loyal, part of Cellular Longevity Inc, San Francisco, California, United States; University of CA, Davis, Davis, California, United States; Cellular Longevity Inc, San Francisco, California, United States; Cellular Longevity Inc, San Francisco, California, United States; Cellular Longevity Inc, San Francisco, California, United States; Cellular Longevity Inc, San Francisco, California, United States; Cellular Longevity Inc, San Francisco, California, United States; Marist College, Poughkeepsie, New York, United States

## Abstract

The companion dog is an emerging model in translational geroscience and offers novel opportunities to investigate aging biology and potential gerotherapeutics. However, there is a scarcity of validated tools and clinical outcome measures to characterize and understand the impact of aging in this translational species. Here we report on a multi-center, cross sectional veterinary clinical study, where we evaluated a clinical questionnaire (Canine Frailty Index; CFI; Banzato et al., 2019) to assess frailty and an owner assessment tool (VetMetrica HRQL) to evaluate HRQL in 451 adult companion dogs. Results support evidence of validity for the tools by confirming expectations that frailty and HRQL deteriorate with age. CFI scores were significantly higher (higher frailty) and HRQL scores significantly lower (worse HRQL) in old dogs (≥ 7 years of age) compared to young dogs (≥ 2 and < 6 years of age). Body size (small < 25lbs or large > 50lbs) was not associated with CFI or total HRQL score. However, older, larger dogs showed faster age-related decline in HRQL scores specific to owner-reported activity and comfort. Findings suggest that the clinician-assessed CFI and owner-reported VetMetrica HRQL may be useful tools to evaluate two determinants of healthspan in dogs: the accumulation of frailty and the progressive decline in quality of life. Establishing validated tools that operationalize the assessment of canine healthspan is critical for the linking pathophysiological mechanisms to aging phenotypes in the companion dog and for accelerating the development of gerotherapeutics that benefit both human and veterinary medicine.

